# Accuracy of CT- vs. Fluoroscopic-Guided Biopsy in Spinal Lesions

**DOI:** 10.3390/jcm15103727

**Published:** 2026-05-12

**Authors:** Sebastian G. Walter, Joline S. Schwan, Thaer Ali, Lioba Bürvenich, Vincent J. Heck, Philipp Rauen, Wolfram Weschenfelder, Sonja Häckel, Nikolaus Kernich

**Affiliations:** 1 Department for Orthopaedic Surgery, Trauma Surgery and Plastic-Aesthetic Surgery, Faculty of Medicine and University Hospital Cologne, University of Cologne, 50937 Cologne, Germanyvincent.heck@uk-koeln.de (V.J.H.); nikolaus.kernich@uk-koeln.de (N.K.); 2 Department for Orthopedic Surgery and Traumatology, Faculty of Medicine, University of Bonn, 53127 Bonn, Germanylioba.buervenich@ukbonn.de (L.B.); 3 Department for Radiology, Faculty of Medicine, University Hospital Cologne, University of Cologne, 50937 Cologne, Germany; philip.rauen@ukkoeln.de; 4 Department of Trauma, Hand and Reconstructive Surgery and Orthopaedics, University Hospital Jena, 07747 Jena, Germany; wolfram.weschenfelder@med.uni-jena.de; 5 Department of Orthopaedic Surgery and Traumatology, Inselspital, Bern University Hospital, University of Bern, 3010 Bern, Switzerland; sonja.haeckel@insel.ch

**Keywords:** spine, tumor, spinal lesion, biopsy, CT-guided, fluoroscopy

## Abstract

**Background**: The rising incidence of vertebral body fractures, vertebral infections and metastatic disease increases the need for diagnostic modalities with high specificity. Biopsy remains essential, yet comparative data on CT-guided versus intraoperative percutaneous fluoroscopy-guided biopsy are limited. **Methods**: This retrospective study compared two cohorts biopsied for spinal lesions between April 2015 and April 2024: CT-guided biopsy (*n* = 62), and intraoperative percutaneous biopsy (*n* = 154). Groups were analyzed for demographic and clinical characteristics, and diagnostic yield was defined by the conclusiveness of the primary biopsy; statistical comparisons were performed using Fisher’s exact test. **Results**: CT-guided biopsy yielded conclusive results in 46 of 62 cases (74.2%), whereas intraoperative, fluoroscopy-guided biopsy was conclusive in 41 of 154 cases (26.6%), representing a statistically significant difference (*p* < 0.001). In analogy, propensity score matching (1:1) resulted in similar significant (*p* < 0.001) results (CT-guided group vs. intraoperative, fluoroscopy-guided group: 86.7% vs. 35.6%) **Conclusions**: CT-guided biopsy demonstrated a substantially higher rate of conclusive results compared with intraoperative biopsy in this cohort. Further studies with larger and more balanced cohorts are needed to strengthen clinical recommendations.

## 1. Introduction

Vertebral body fractures constitute a major clinical and socioeconomic challenge worldwide, and they represent one of the most frequent osteoporotic fracture types, with increasing incidence over the past decades [1]. For example, in Germany in 2019, they accounted for approximately 13% of all fractures [2], with more than 87,000 cervical, thoracic, and lumbar vertebral fractures being recorded. Incidence increases markedly with age, reflecting demographic changes and the rising prevalence of osteoporosis and malignancy. While high-energy trauma predominates as the underlying cause in younger individuals [3], osteoporotic-insufficiency fractures and malignant spinal involvement are the leading etiologies in older patients [4]. Given that approximately 885,000 individuals in Germany each year are newly diagnosed with osteoporosis [5], and nearly 500,000 with cancer [6], a substantial and growing population is at risk for vertebral fractures and malignant lesions and associated complications. The aging of the population further amplifies this relevance, with projections indicating a marked increase in life expectancy over the coming decades [7], thereby expanding the population at risk for osteoporosis-related, cancer-related and infectious spinal pathology [8].

From a clinical perspective, differentiating between benign osteoporotic fractures, infectious spondylodiscitis (vertebral osteomyelitis), and malignant spinal lesions is essential, as their respective management strategies, prognostic implications and therapeutic urgency differ substantially [9]. Although imaging modalities such as magnetic resonance imaging (MRI), computed tomography (CT), and positron emission tomography (PET) deliver significantly improved valuable diagnostic information, radiological findings alone may be insufficient to reliably distinguish between benign and malignant lesions, particularly in cases with atypical morphology, early metastatic disease or overlapping imaging characteristics. Recent studies have emphasized that even sophisticated MRI protocols may yield indeterminate results in a subset of patients [10], necessitating histopathological confirmation. Therefore, the material obtained in biopsy, and the quality of this material, are decisive for pathological assessment and precise classification of lesions [11].

Percutaneous, fluoroscopy-guided biopsy continues to be regarded as the gold standard for achieving a definitive diagnosis in patients presenting with indeterminate and suspicious vertebral lesions. Such lesions are typically identified during pre-interventional, contrast-enhanced MRI when they demonstrate atypical morphological characteristics and/or exhibit abnormal patterns of contrast enhancement that preclude precise classification through imaging alone. In these situations, imaging findings are insufficient to exactly define the underlying neoplastic etiology or characterize the pathogen responsible for infection. Therefore, obtaining an adequate tissue sample is of critical importance. High-quality tissue acquisition ensures improved diagnostic sensitivity and specificity, thereby reducing the risk of inconclusive or misleading results. Ultimately, the reliability of the biopsy directly impacts clinical decision-making, guiding the selection of appropriate therapeutic strategies and influencing patient outcomes.

Two principal biopsy techniques are commonly employed in clinical practice: CT-guided percutaneous biopsy and (intra-)operative percutaneous biopsy performed during surgical procedures such as kyphoplasty or vertebroplasty.

CT-guided biopsy is widely regarded as a precise and minimally invasive approach offering accurate needle placement under real-time imaging control. Several recent systematic reviews [12] and large-cohort studies [13] have reported high diagnostic yields and favorable safety profiles across a broad spectrum of musculoskeletal lesions [14], supporting the role of CT-guided biopsy as a safe and effective diagnostic procedure. However, concerns remain regarding sample representativeness, particularly when biopsies are performed without specific radiological suspicion [15].

In parallel, intraoperative biopsy offers the advantage of tissue acquisition during a therapeutic intervention, potentially avoiding an additional invasive procedure [16]. However, the diagnostic accuracy and clinical utility of routine intraoperative biopsy both remain subjects of ongoing debate. While some recent studies have reported that intraoperative biopsies may detect unexpected malignancy in a subset of patients [17] and prospective studies have specifically advocated for routine biopsies in every patient with osteoporotic vertebral compression fractures [18], others have reported relatively low diagnostic yields and questioned routine biopsy use in the absence of specific clinical or radiological suspicion, due to poor cost-effectiveness and a lack of clinical necessity [19]. Although there is a growing body of literature in this field, existing studies focus primarily on diagnostic yields or safety outcomes. Direct comparative analyses between CT-guided and intraoperative biopsy techniques with a specific focus on diagnostic specificity and clinically meaningful outcomes remain scarce, particularly in European healthcare settings.

Furthermore, recent publications have emphasized the need for more selective biopsy strategies based on patient- and lesion-related variables like risk factors, imaging characteristics, and clinical context [20]. Emerging evidence shows that factors such as patient age, known prior malignancy, fracture morphology, lesion localization, number of sampled cores and imaging features may influence biopsy yield and diagnostic accuracy [21]. Nonetheless, robust comparative data evaluating how these variables interact with different biopsy techniques are limited.

In light of these considerations, the present study aims to compare the diagnostic specificity and overall clinical value of CT-guided percutaneous biopsy and intraoperative percutaneous biopsy in patients with vertebral body lesions.

## 2. Materials and Methods

This retrospective investigation was conducted at the University Hospital Cologne, Germany. All patients who underwent either CT-guided percutaneous vertebral biopsy or intraoperative percutaneous biopsy between April 2015 and April 2024 were screened for eligibility.

The requirement for informed consent was waived due to the retrospective nature of the study. The study was conducted in accordance with the Declaration of Helsinki, and the protocol was approved by the Ethics Committee of the University Hospital of Cologne (approval code: 20-1643) in November 2020. For transparency a STROBE statement protocol was performed (Supplementary Materials).

Patients were eligible if they underwent biopsy of a vertebral body lesion either for diagnostic clarification of an unclear vertebral lesion or intraoperatively during a therapeutic intervention with suspicion for malignancy or infection objectified by pre-interventional MRI assessment (contrast-enhanced MRI with atypical morphologic findings and/or increased contrast enhancement). Exclusion criteria were incomplete or missing medical records, biopsies performed as part of open surgical procedures, biopsies targeting non-vertebral anatomical sites (e.g., scapula, pelvis), and revision biopsies.

CT-guided biopsies were performed under sterile conditions using CT imaging for real-time needle guidance. Intraoperative biopsies were performed during vertebral augmentation procedures such as kyphoplasty. Typically, at least 3 biopsy cores were obtained during each procedure. Tissue samples were obtained prior to cement application. Due to the retrospective nature of this study, factors as needle type, gauge sizes, numbers of samples taken or the operating surgeon could not be standardized. All tissue samples were processed according to institutional pathology protocols. Histological examination was performed by certified pathologists. Again, standardization of pathological examinations was not possible due to the retrospective character of this investigation.

Data were extracted from electronic medical records from the inter-departmental computer system of the University Hospital Cologne, and were collected using a predefined data collection sheet designed with Microsoft Excel. Data collected included patient demographics (age, sex), biopsy date, spinal level and region, biopsy technique (CT-guided vs. intraoperative), type of surgical intervention if applicable, comorbidities, histopathological and microbiological findings, postoperative discharge summaries, and final diagnosis. Comorbidities were assessed based on the most recent medical report available.

Biopsies were classified as conclusive if they yielded a definitive etiology of the vertebral lesion in the first attempt. A diagnosis of tumor was assigned when histopathology demonstrated unequivocal neoplastic cells. Infection was diagnosed based on histological evidence of acute or chronic inflammation in conjunction with positive microbiological findings. Osteoporosis was accepted as the final diagnosis if explicitly stated in the pathological report. If the pathological diagnosis differed substantially from the discharge diagnosis, the biopsy was classified as non-conclusive. Biopsies were also considered non-representative when the pathological report was not conclusive and a second biopsy was recommended or the specimen was declared non-representative.

All data were compiled using Microsoft Excel (Microsoft Corp., Redmond, WA, USA) and statistical analysis was performed using SPSS version 30 (IBM Corp., Armonk, NY, USA). Descriptive statistics were calculated for all variables. Continuous variables were tested for normal distribution using the Kolmogorov–Smirnov test. Group comparisons for categorical variables were conducted using Fisher’s exact test or the Fisher–Freeman–Halton test for multi-category comparisons, as appropriate. Statistical significance was set at *p* < 0.05. To exclude confounding due to imbalanced baseline characteristics, additional propensity score matching was performed. Matching included age, gender, the vertebral height of the lesion, and previously diagnosed malignancies.

## 3. Results

**Figure 1:**
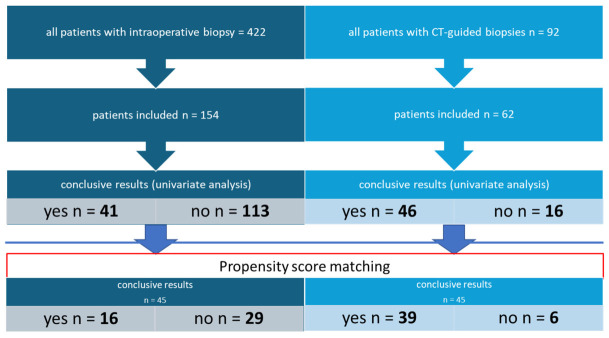
Patient selection within both cohorts, with univariate analysis and propensity score matching analysis.

A total of 514 patients were screened, including 422 patients in the intraoperative biopsy group and 92 in the CT-guided group. After excluding patients who did not meet the predefined inclusion and exclusion criteria (Table 1), 216 patients were included in the final analysis (Figure 1). The final cohort comprised 112 women (51.9%) and 104 men (48.1%). The mean age of the groups differed by 12.1 years. Additionally, propensity score matching (ratio 1:1) was performed; this resulted in two cohorts of 45 patients, each with similar baseline characteristics (Table 2). Retrospective analysis revealed that lesion size in all cases was at least 1 × 1 × 1 cm.

In the intraoperative group (*n* = 154), 41 biopsies (26.6%) were conclusive and 113 (73.4%) non-conclusive. In the CT-guided group (*n* = 62), 46 biopsies (72.4%) were conclusive and 16 (25.8%) non-conclusive. Therefore, an absolute risk difference of 47.6% (*p* < 0.001, OR 7.92, 95% CI 4.05–15.52) could be assumed. Among the 41 conclusive intraoperative biopsies, 37 revealed tumors (90.2%) and three revealed infections (7.3%), while in one patient, both tumor and osteoporosis were present, and in another, osteoporosis was diagnosed as the only finding.

The segment distribution of vertebral lesions showed no significant (*p* > 0.05) difference between the intraoperative group and the CT-guided group (thoracic spinal column 38,3% vs. 33,9%; lumbar spinal column 49,4% vs. 51,6%).

When propensity score matching (1:1 ratio) was performed, there were 45 patients in each group. While in the CT-guided group 86.7% (*n* = 39) of biopsies were conclusive, this was the case in only 35.6% (*n* = 16) of the intraoperative, fluoroscopic-guided group (*p* < 0.001; OR 11.78, 95% CI 4.11–33.81).

In this cohort, re-biopsy was recommended by pathologists for the intraoperative group in 15 cases (33.3%) while in the CT-guided group this accounted for one case (2.2%) only.

In the CT-guided group, 36 conclusive biopsies diagnosed tumors (80.0%), and three identified infections (6.7%).

**Figure 2:**
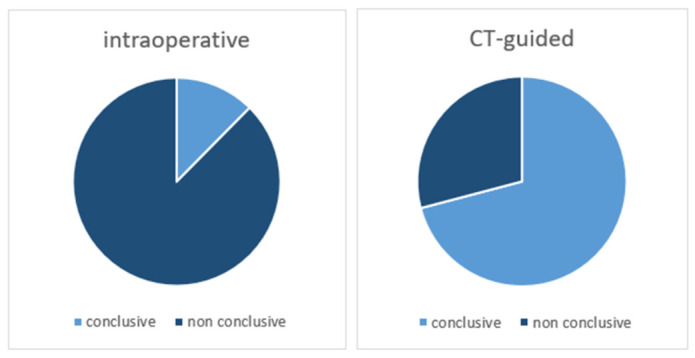
Distribution of results in both cohorts (*n* = 45 each).

Fisher’s exact test demonstrated a statistically significant difference (*p* < 0.001) between the compared techniques with regard to conclusive biopsy outcomes (Table 3, Figure 2). CT-guided biopsy was associated with significantly higher odds of obtaining a conclusive diagnosis, compared to intraoperative biopsy (OR 11.78, 95% CI 4.11–33.81; *p* < 0.001).

## 4. Discussion

This study indicates that CT-guided biopsies are associated with a significantly higher likelihood of yielding a conclusive result compared to intraoperative, fluoroscopy-guided biopsies. This conclusion stands in contrast to those of other published studies that have found no significant difference between fluoroscopic and CT-guided biopsies [22]. To the best of our knowledge, there is so far only very limited evidence on this specific topic.

Both biopsy techniques are influenced not only by the performing physician but also by patient-specific factors. Potential determinants—such as operator experience, or lesion depth—were not evaluated in this study. Additionally, the quantity and quality of biopsy material may be affected by the choice of instruments, such as needle type [23]. Furthermore, this study is retrospective in nature. Parameters such as the operating physician and the instruments used were not standardized. Furthermore, the relatively small sample size, drawn from a single institution, limits the generalizability of the findings.

Diagnostic procedures must also be considered within a broader context. CT-guided biopsy exposes both patients and medical staff to ionizing radiation, necessitating additional safety measures [24]. Intraoperative biopsies are often performed during surgical procedures such as kyphoplasty, which may be indicated due to instability caused by the lesion [25]. When vertebral augmentation is not immediately necessary, CT-guided biopsy offers a safe and less invasive option [26]. Higher rates of conclusive biopsy results in the CT-guided group can be explained by the three-dimensional visualization of the lesion and the confirmation of the needle’s trajectory right into the lesion.

It must be acknowledged that the retrospective design of the present work does leave certain aspects unresolved. Discrepancies between biopsy site descriptions in operative and pathology reports were observed. Follow-up results from repeat biopsies recommended by pathology were sometimes missing, preventing confirmation or disproof of the initial diagnosis. In some cases, the final diagnosis, particularly diagnosis of osteoporosis, was established clinically on the basis of overall patient assessment, rather than on histological findings. Such an integrated view of a patient cannot be captured in a retrospective study, potentially leading to classification of some biopsies as non-conclusive, even in cases when subsequent adequate treatment results confirmed a suspected diagnosis.

The study may further be limited by the relatively long observation interval of about ten years, which implies different operators with different levels of experience and the possibility of different needle types. However, it is one of the first studies to address this specific research question, and further prospective investigations are desirable.

## 5. Conclusions

This retrospective study found a significantly higher diagnostic yield from CT-guided percutaneous biopsy compared with intraoperative percutaneous biopsy for vertebral body lesions. CT-guided biopsy yielded conclusive results in the majority of cases, whereas intraoperative biopsy was associated with a substantially higher proportion of non-conclusive findings. These results highlight the added diagnostic value of CT guidance in achieving representative tissue samples, particularly in patients with unclear vertebral lesions.

By directly comparing two commonly used biopsy techniques within the realistic cohort of a tertiary care center, this study provides evidence to support clinical decision-making and optimize diagnostic pathways for malignancy or infection suspicious vertebral lesions.

Several limitations should be acknowledged. The retrospective study design and the unequal group sizes introduce potential selection bias. The absence of standardized biopsy protocols and the single-center setting may further limit the generalizability of the results. Additionally, potential confounders such as lesion size, biopsy needle type, and operator experience were not systematically analyzed.

Future research should focus on prospective, multicenter studies with standardized procedural protocols and balanced cohorts to validate these findings. Further studies integrating clinical, radiological, and procedural variables into predictive models may enable more precise, risk-adapted biopsy strategies, thereby improving diagnostic accuracy, reducing repeat procedures, and optimizing patient care.

## Figures and Tables

**Table 1:** Inclusion and exclusion criteria.

Inclusion Criteria	Exclusion Criteria
Age > 18 years	Age < 18 years
Pre-interventional MRI	Incomplete medical records
Vertebral lesion suspicious for malignancy or infection	Extra-vertebral tumor location
	Biopsy during open spine surgery

**Table 2:** Baseline characteristics after propensity score matching. For simplification the spine was sectionized into cervical (1), thoracic (2), lumbar (3) and sacral (4) spine.

Variable	Intraoperative (*n* = 45)	CT-Guided (*n* = 45)
Age (mean SD)	64.09 (±14.10)	64.29 (±11.10)
Female (total)	21	20
Male (total)	24	25
Positive tumor anamnesis	35	33
Vertebral section	1 = 0 2 = 15 3 = 22 4 = 8	1 = 4 2 = 17 3 = 24 4 = 0

**Table 3:** Overview of overall conclusiveness of biopsies depending on biopsy technique in a cohort balanced for baseline characteristics (age, gender, vertebral section, positive tumor anamnesis). * *p*-value was regarded significant for <0.05.

Propensity-Matched Results				
	Conclusive (***n***, %)	Non-Conclusive (***n***, %)	OR (95% CI)	***p***-Value *
Intraoperative	16 (35.6%)	29 (64.4%)	Reference	
CT-guided	39 (86.7%)	6 (13.3%)	11.78 (4.11–33.81)	<0.001
Total	55 (61.1%)	35 (38.9%)		

## Data Availability

Access to data will be granted upon request to the corresponding author.

## Supplementary Materials

The following supporting information can be downloaded at: https://www.mdpi.com/article/10.3390/jcm15103727/s1, Table S1: STROBE Statement—Checklist of items that should be included in reports of cohort studies.

## References

[1] Niu H.-G., Hu Y., Gong Y.-K., Hu G.-K., Ye G.-Q., Gao W.-S. (2025). Trends in prevalence of spine fractures and risk factors in spine fractures among US adults, 1999–2018. Sci. Rep..

[2] Rupp M., Walter N., Pfeifer C., Lang S., Kerschbaum M., Krutsch W., Baumann F., Alt V. (2021). The Incidence of Fractures Among the Adult Population of Germany–an Analysis From 2009 through 2019. Dtsch. Arztebl. Int..

[3] Farr J.N., Melton L.J., Achenbach S.J., Atkinson E.J., Khosla S., Amin S. (2017). Fracture Incidence and Characteristics in Young Adults Aged 18 to 49 Years: A Population-Based Study. J. Bone Miner. Res..

[4] Spinnato P., Bazzocchi A., Facchini G., Filonzi G., Nanni C., Rambaldi I., Rimondi E., Fanti S., Albisinni U. (2018). Vertebral Fractures of Unknown Origin: Role of Computed Tomography-Guided Biopsy. Int. J. Spine Surg..

[5] Hadji P., Klein S., Gothe H., Häussler B., Kless T., Schmidt T., Steinle T., Verheyen F., Linder R. (2013). The epidemiology of osteoporosis—Bone Evaluation Study (BEST): An analysis of routine health insurance data. Dtsch. Arztebl. Int..

[6] Robert Koch Institut (2024). Krebs Gesamt. https://www.krebsdaten.de/Krebs/DE/Content/Krebsarten/Krebs_gesamt/krebs_gesamt_node.html.

[7] Statistisches Bundesamt (2025). 15. Koordinierte Bevölkerungsvorausrechnung. https://www.destatis.de/DE/Themen/Gesellschaft-Umwelt/Bevoelkerung/Bevoelkerungsvorausberechnung/begleitheft.html?nn=238640#lebenserwartung11.08.

[8] Heck V.J., Prasse T., Klug K., Vinas-Rios J.M., Oikonomidis S., Klug A., Kernich N., Weber M., von der Höh N., Lenz M. (2024). The projected increase of vertebral osteomyelitis in Germany implies a demanding challenge for future healthcare management of aging populations. Infection.

[9] Liang Y., Liu P., Jiang L.B., Wang H., Hu A., Zhou X., Li X., Lin H., Wu D., Dong J. (2019). Value of CT-guided Core Needle Biopsy in Diagnosing Spinal Lesions: A Comparison Study. Orthop. Surg..

[10] Tarigan V.N., Kusumaningtyas N., Supit N., Sanjaya E., Chandra M., Sulay C.B.H., Octavius G.S. (2025). An Updated Systematic Review and Meta-Analysis of Diagnostic Accuracy of Dynamic Contrast Enhancement and Diffusion-Weighted MRI in Differentiating Benign and Malignant Non-Mass Enhancement Lesions. J. Clin. Med..

[11] Adyanthaya S., Jose M. (2013). Quality and safety aspects in histopathology laboratory. J. Oral Maxillofac. Pathol..

[12] Michalopoulos G.D., Yolcu Y.U., Ghaith A.K., Alvi M.A., Carr C.M., Bydon M. (2021). Diagnostic yield, accuracy, and complication rate of CT-guided biopsy for spinal lesions: A systematic review and meta-analysis. J. Neurointerv. Surg..

[13] Winkler W.L., Baker J.C., Tomasian A., Velde T.L.V., Hillen T.J., Luo C., Imaoka R., Dettorre G.M., Jennings J.W. (2024). Diagnostic efficacy of image-guided core needle biopsy of suspected malignant osseous lesions: A retrospective cohort study from a single academic institution. Eur. Radiol..

[14] Yang S.Y., Oh E., Kwon J.W., Kim H.S. (2018). Percutaneous Image-Guided Spinal Lesion Biopsies: Factors Affecting Higher Diagnostic Yield. AJR Am. J. Roentgenol..

[15] Kim W., Sun K., Kung J.W., Wu J.S. (2023). CT-Guided Core Needle Biopsy of Nonspinal Bone Lesions: Comparison of Occult and Visible Bone Lesions. AJR Am. J. Roentgenol..

[16] Li Q., Hua S., Wang C., Cai S., Zhang J. (2014). The value of routine biopsy during percutaneous kyphoplasty for vertebral compression fractures. PLoS ONE.

[17] Osterhoff G., Scheyerer M.J., Spiegl U.J.A., Schnake K.J. (2023). The role of routine transpedicular biopsies during kyphoplasty or vertebroplasty for vertebral compression fractures in the detection of malignant diseases: A systematic review. Arch. Orthop. Trauma Surg..

[18] Zhang Y., Zhao M., Tao L., Zhang Y., Li K., Guo S., Su J., Hai Y., Liu Y., Su Q. (2025). Value the importance of routine biopsy during vertebral augmentation: A prospective observational study of one hundred and forty one patients. Int. Orthop..

[19] Hershkovich O., Bayley E., Rudik O., Alexandrovsky V., Friedlander A., Daglen E., Lotan R. (2020). Cost-Benefit Analysis of Routine Bone Biopsy During Augmentation of Osteoporotic Vertebral Compression Fractures. Spine.

[20] Zhu Y., Yang K., Wang C., Fan Y., Wu X., He S., Gu G. (2025). Towards optimized biopsy use in vertebral compression fractures: Integrating risk assessment for better clinical decision-making. Int. Orthop..

[21] Bulut H., Lam C., Sheth V., Ali I., Tsagkaris C., Jones M., Botchu R., Errani C., Hamzaoglu A., Ozkan K. (2025). Evaluating the Outcomes of Vertebral Biopsies Performed in Osteoporotic Vertebral Fractures: A Systematic Review and Meta-Analysis. Osteology.

[22] Oka M., Suzuki A., Terai H., Kato M., Toyoda H., Takahashi S., Tamai K., Nakamura H. (2023). Factors Predicting the Final Diagnosis in Image-Guided Percutaneous Needle Biopsy for Suspected Spinal Tumors. J. Clin. Med..

[23] Zakaria Mohamad Z., Rahim A.A., Kow R.Y., Karupiah R.K., Zainal Abidin N.A., Mohamad F. (2022). Diagnostic Accuracy and Adequacy of Computed Tomography Versus Fluoroscopy-Guided Percutaneous Transpedicular Biopsy of Spinal Lesions. Cureus.

[24] Kloeckner R., Santos D.P.D., Schneider J., Kara L., Dueber C., Pitton M.B. (2013). Radiation exposure in CT-guided interventions. Eur. J. Radiol..

[25] Alsoof D., Anderson G., McDonald C.L., Basques B., Kuris E., Daniels A.H. (2022). Diagnosis and Management of Vertebral Compression Fracture. Am. J. Med..

[26] Singh D.K., Kumar N., Nayak B.K., Jaiswal B., Tomar S., Mittal M.K., Bajaj S.K. (2020). Approach-based techniques of CT-guided percutaneous vertebral biopsy. Diagn. Interv. Radiol..

