# Supplementary material for: Accuracy of CT- vs. Fluoroscopic-Guided Biopsy in Spinal Lesions
Source: J Clin Med. 2026 May 12;15(10):3727. doi: 10.3390/jcm15103727 (PMC13208035; doi:10.3390/jcm15103727)
Supplement: Supplementary file 1 [file jcm-15-03727-s001.zip › jcm-4208292-supplementary.pdf]

Table S1. STROBE Statement—Checklist of items that should be included in reports of cohort studies.

	Item No.	Recommendation
**Title and Abstract**	1	(*a*) Indicate the study's design with a commonly used term in the title or the abstract
		(*b*) Provide in the abstract an informative and balanced summary of what was done and what was found
**Introduction**		
Introduction, Paragraph 1	2	Explain the scientific background and rationale for the investigation being reported
Introduction, Paragraphs 2–5	3	State specific objectives, including any prespecified hypotheses
**Methods**		
Materials and Methods, Paragraph 1	4	Present key elements of study design early in the paper
Materials and Methods, Paragraphs 1,3,4	5	Describe the setting, locations, and relevant dates, including periods of recruitment, exposure, follow-up, and data collection
Materials and Methods, Table 1, Paragraph 4	6	(*a*) Give the eligibility criteria, and the sources and methods of selection of participants. Describe methods of follow-up
		(*b*) For matched studies, give matching criteria and number of exposed and unexposed
Materials and Methods, Paragraph 4	7	Clearly define all outcomes, exposures, predictors, potential confounders, and effect modifiers. Give diagnostic criteria, if applicable
Materials and Methods, Paragraph 4	8 *	For each variable of interest, give sources of data and details of methods of assessment (measurement). Describe comparability of assessment methods if there is more than one group
Materials and Methods, Paragraph 4	9	Describe any efforts to address potential sources of bias
Results, Figure 1, Paragraph 1	10	Explain how the study size was arrived at
Materials and Methods, Paragraph 5, Results, Paragraph 1, Figure 1	11	Explain how quantitative variables were handled in the analyses. If applicable, describe which groupings were chosen and why
Materials and Methods, Paragraphs 4, 5	12	(*a*) Describe all statistical methods, including those used to control for confounding
		(*b*) Describe any methods used to examine subgroups and interactions
		(*c*) Explain how missing data were addressed
		(*d*) If applicable, explain how loss to follow-up was addressed
		(*e*) Describe any sensitivity analyses
**Results**		
Results, Figure 1, Paragraph 1	13 *	(a) Report numbers of individuals at each stage of study, e.g., numbers potentially eligible, examined for eligibility, confirmed eligible, included in the study, completing follow-up, and analyzed
		(b) Give reasons for non-participation at each stage
		(c) Consider use of a flow diagram
Results, Paragraph 1, Table 2	14 *	(a) Give characteristics of study participants (e.g., demographic, clinical, social) and information on exposures and potential confounders
		(b) Indicate number of participants with missing data for each variable of interest
		(c) Summarize follow-up time (e.g., average and total amount)
Results, Figures 1,2, Table 3, Paragraphs 2–6	15 *	Report numbers of outcome events or summary measures over time
Results, Table 3, Paragraphs 2, 4	16	(*a*) Give unadjusted estimates and, if applicable, confounder-adjusted estimates and their precision (e.g., 95% confidence interval). Make clear which confounders were adjusted for and why they were included
		(*b*) Report category boundaries when continuous variables were categorized
		(*c*) If relevant, consider translating estimates of relative risk into absolute risk for a meaningful time period

Results, Paragraph 2	17	Report other analyses done—e.g., analyses of subgroups and interactions, and sensitivity analyses
**Discussion**		
Discussion, Paragraph 1	18	Summarize key results with reference to study objectives
Discussion, Paragraphs 2–5	19	Discuss limitations of the study, taking into account sources of potential bias or imprecision. Discuss both the direction and magnitude of any potential bias
Discussion, Paragraph 3, Conclusion	20	Give a cautious overall interpretation of results considering objectives, limitations, multiplicity of analyses, results from similar studies, and other relevant evidence
Conclusion	21	Discuss the generalizability (external validity) of the study results
**Other information**		
Funding	22	Give the source of funding and the role of the funders for the present study and, if applicable, for the original study on which the present article is based

* Give information separately for exposed and unexposed groups. **Note:** An Explanation and Elaboration article discusses each checklist item and gives the methodological background and published examples of transparent reporting. The STROBE checklist is best used in conjunction with this article (freely available on the websites of PLoS Medicine, at http://www.plosmedicine.org/ (20 February 2026); the Annals of Internal Medicine, at http://www.annals.org/ (20 February 2026); and of Epidemiology, at http://www.epidem.com/ (20/02/2026)). Information on the STROBE Initiative is available at http://www.strobe-statement.org. (20 February 2026)
